# Virological and immunological determinants of hepatitis E virus infection outcomes

**DOI:** 10.1016/j.coviro.2025.101491

**Published:** 2025-09-19

**Authors:** Xinjia Wang, Lu Bian, Eun Hee Ha, Lei Wang, Kyle O’Shaughnessy, Yifei Zheng, Zhuoying Feng, Wei Bo Chen, Xianfang Wu

**Affiliations:** 1 Department of Infection Biology, Lerner Research Institute, Cleveland Clinic, Cleveland, OH 44195, United States of America; 2 Department of Genetics and Genome Sciences, School of Medicine, Case Western Reserve University, Cleveland, OH 44195, United States of America; 3 Cleveland Clinic College of Medicine at Case Western Reserve University, Cleveland, OH 44195, United States of America

## Abstract

Hepatitis E virus (HEV) is a leading cause of acute viral hepatitis worldwide, especially in regions with limited sanitation infrastructure. Unlike hepatitis B virus (HBV) and hepatitis C virus (HCV) — which frequently progress to chronic infections associated with cirrhosis and hepatocellular carcinoma — HEV typically leads to acute and self-resolving infection in immunocompetent individuals. This review explores the mechanistic underpinnings that distinguish HEV’s clinical trajectory from HBV and HCV, focusing on viral genome organization, replication strategies, immune evasion tactics, and host immune responses. Understanding these factors provides critical insights into the determinants of viral clearance versus persistence and may guide future vaccine and antiviral development.

## Introduction

Acute viral hepatitis can be caused by several hepatotropic viruses, among which hepatitis B virus (HBV), hepatitis C virus (HCV), and hepatitis E virus (HEV) are of particular global importance due to their substantial disease burden [1]. While all three can cause acute liver inflammation, their long-term clinical outcomes vary significantly. Most adults infected with HBV successfully clear the virus; however, approximately 5% develop chronic infection, a rate that rises to nearly 90% for those infected perinatally or during early childhood [2]. Chronic HBV infection can lead to progressive liver fibrosis, cirrhosis, and hepatocellular carcinoma (HCC), especially in individuals with high viral loads or prolonged immune tolerance [3]. HCV differs in that it becomes chronic in the majority (~70–85%) of infected individuals, placing them at significant risk for fibrosis, cirrhosis, and HCC if left untreated [4]. In contrast, HEV typically causes a self-limited acute infection in immunocompetent hosts, with chronicity being rare [5]. Nevertheless, HEV is responsible for an estimated 20 million infections annually, including over 3 million symptomatic cases and approximately 44 000 deaths — primarily due to fulminant hepatitis in pregnant women and individuals with pre-existing liver disease [6,7].

These differences in clinical outcomes are further shaped by the distinct epidemiological patterns and genotypic characteristics of HEV, which influence its transmission routes and disease manifestations. HEV genotypes vary in their epidemiological and clinical profiles. Genotypes 1 and 2 are restricted to humans and are mainly associated with waterborne outbreaks in developing regions. Genotypes 3 and 4 are zoonotic, endemic in industrialized countries, and transmitted via consumption of undercooked pork or game meat [8]. Chronic HEV infection is observed almost exclusively with genotype 3 and occurs primarily in immunosuppressed individuals, such as solid organ transplant recipients, patients receiving chemotherapy, and people living with HIV/AIDS [9-11].

This marked divergence in clinical outcomes — chronic infection with HBV and HCV versus spontaneous clearance in HEV — raises critical questions about the viral and host determinants that govern infection resolution versus persistence (Table 1). In this review, we explore key differences in virology, host–virus interactions, and immune evasion strategies that may underlie HEV’s comparatively benign clinical course. Understanding why HEV typically causes a self-limiting infection in immunocompetent individuals is not only biologically intriguing but also clinically significant. Elucidating the viral and host factors that contribute to HEV clearance may uncover fundamental principles of effective immune control over hepatotropic viruses. These insights could inform the development of new therapeutic strategies aimed at restoring immune competence in chronic HBV and HCV infections or preventing their progression to severe liver complications such as cirrhosis and HCC.

## Virological determinants

HEV is a positive-sense, single-stranded RNA virus approximately 7.2 kb in length and a member of the *Hepeviridae* family [8,12]. Its genome contains three well-characterized open reading frames (ORFs): ORF1, ORF2, and ORF3. ORF1 encodes a large nonstructural polyprotein comprising multiple enzymatic domains essential for viral replication, including methyltransferase, protease, helicase, and RNA-dependent RNA polymerase. ORF2 encodes the capsid protein, which is responsible for virion assembly, host receptor interaction, and eliciting host immune responses. ORF3 encodes a small multifunctional phosphoprotein that modulates host cell signaling and facilitates viral egress, particularly via the exosomal pathway.

**Figure 1:**
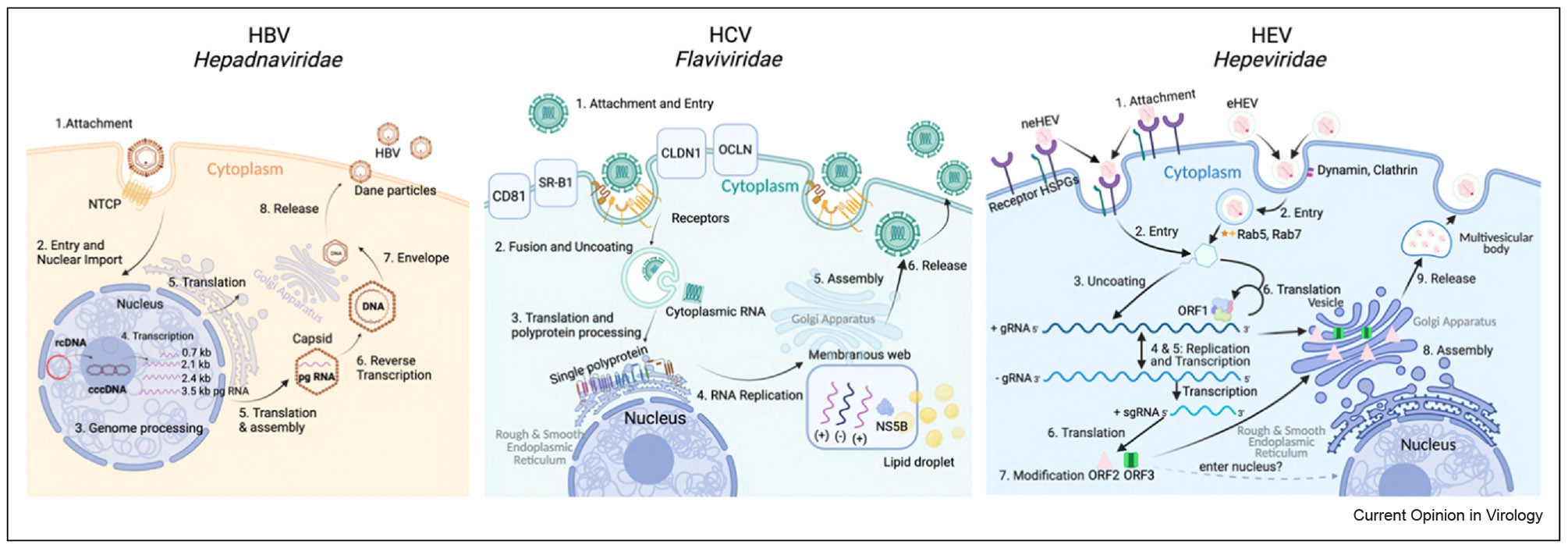
Comparative lifecycles of HBV, HCV, and HEV. Schematic overview of the replication cycles of HBV, HCV, and HEV, highlighting key similarities and differences. HBV requires nuclear import and cccDNA formation with reverse transcription in the cytoplasmic capsid. HCV undergoes cytoplasmic RNA replication within a membranous web, tightly linked to lipid metabolism. HEV replicates entirely in the cytoplasm, producing both quasi-enveloped (eHEV) and nonenveloped (neHEV) virions for release (the figure was generated with Biorender.com).

**Figure 2:**
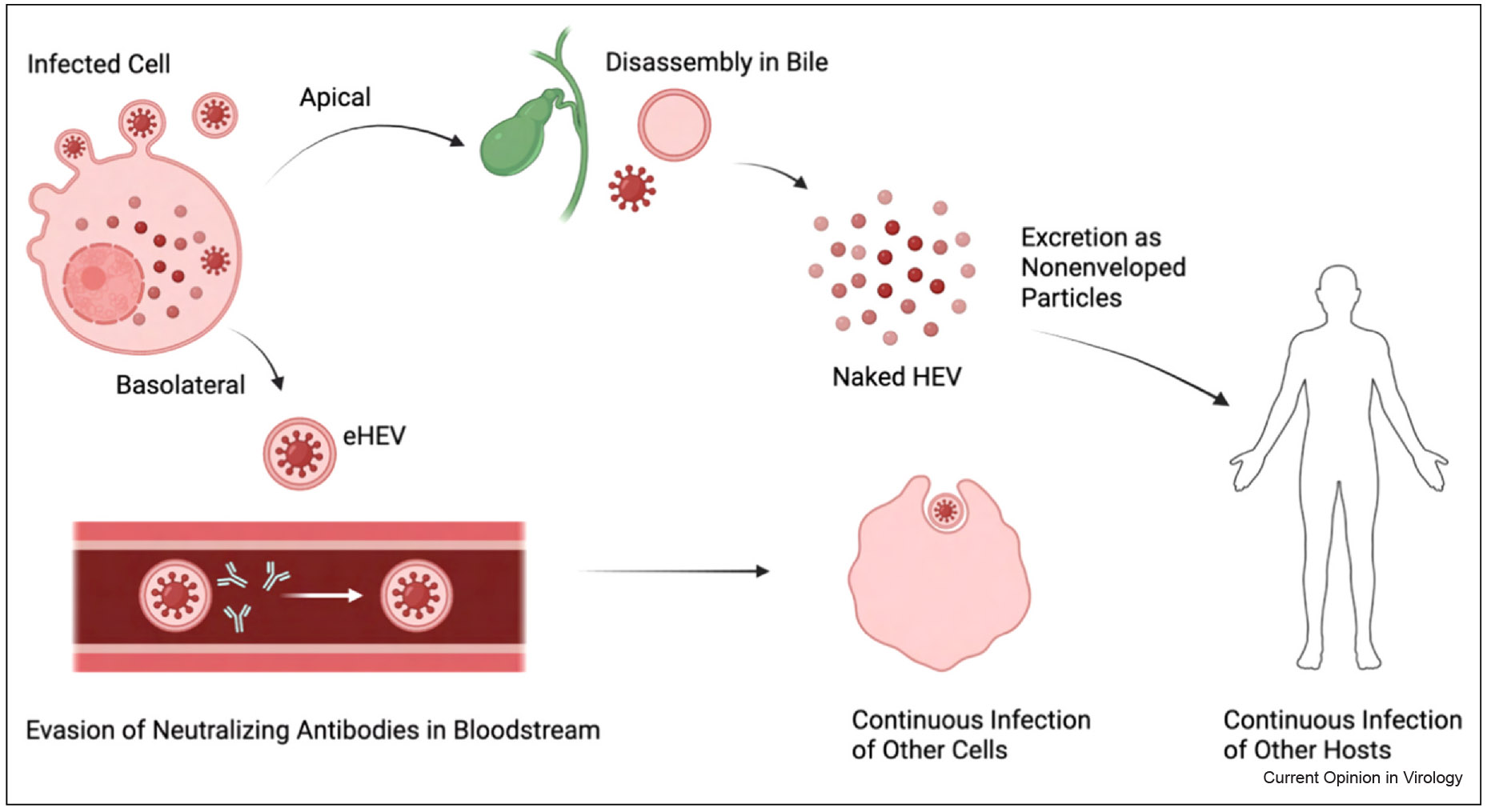
Dual egress pathways and the transmission dynamics of HEV. Newly assembled HEV progeny are released from infected hepatocytes through two distinct cellular routes: the basolateral and apical membranes. Virions exiting via the basolateral side enter the bloodstream cloaked in a host-derived membrane, forming the quasi-enveloped form of the virus (eHEV). This lipid envelope helps the virus evade neutralizing antibodies in circulation, allowing it to persist transiently in the bloodstream and potentially infect naïve cells in other tissues. HEV particles secreted through the apical surface into the bile duct are exposed to the detergent-like environment of bile acids. This exposure strips the envelope from the virions, resulting in nonenveloped, or ‘naked’ HEV particles. These more immunogenic forms are ultimately excreted in the feces and are responsible for fecal–oral transmission (the figure was generated with Biorender.com).

The HEV life cycle is largely cytoplasmic [13]. Upon entry into the host cell, the viral genome is translated to produce the ORF1 polyprotein, which supports RNA replication through a negative-sense intermediate. This intermediate serves as a template for synthesizing both new genomic RNA and a subgenomic RNA encoding ORF2 and ORF3 (Figure 1). Virion assembly occurs in the cytoplasm, and HEV is released either as non-enveloped virions (in bile and feces) or as quasi-enveloped virions (eHEV in the bloodstream, Figure 2) via the multivesicular body (MVB)/exosomal pathway [14,15]. Notably, HEV replication does not involve the host nucleus, DNA intermediates, or chromosomal integration.

### Comparison with HBV

HBV is a partially double-stranded DNA virus that establishes persistent infection in part through the formation of covalently closed circular DNA (cccDNA) within the nucleus of hepatocytes (Figure 1). This stable episomal form serves as a transcriptional reservoir, allowing for prolonged viral gene expression and resistance to antiviral therapy [16]. In contrast to HBV, HEV lacks a DNA stage and does not integrate into the host genome, which greatly limits its ability to establish a chronic infection in immunocompetent hosts (Table 1). HBV also produces large quantities of subviral particles, including HBeAg and HBsAg, which can induce immune tolerance or act as decoys [17]. While HEV does secrete noninfectious capsid proteins similar to those of HBV [18], their immunoregulatory roles appear far less pronounced. Furthermore, HBV utilizes host nuclear transcription machinery and tightly regulates its gene expression through host- and virus-derived factors. HEV, by replicating primarily in the cytoplasm, thereby minimizes these nuclear regulatory interactions [19], which may in turn limit its capacity for long-term persistence.

### Comparison with HCV

HCV is an enveloped, positive-sense RNA virus that, despite lacking a DNA stage, is remarkably proficient at establishing chronic infections [4]. This is largely attributed to its error-prone RNA polymerase, which generates a diverse viral quasispecies population within the host [20,21]. In contrast, HEV appears to maintain a genetically stable genome in untreated individuals [22], likely resulting in a comparatively narrow quasispecies distribution (Figure 1).

This fundamental difference in quasispecies dynamics has important implications for infection outcomes. The role of viral quasispecies diversity in shaping persistence and immune escape has been highlighted in several studies, including a foundational review by Bowen and Walker [23], which proposes that high quasispecies complexity correlates with greater adaptability and persistence within the host. From this perspective, the limited genetic variability of HEV may constrain its ability to evade immune responses, thereby favoring viral clearance in immunocompetent individuals. Conversely, viruses like HCV — with their broad mutant spectra — are more capable of generating immune escape variants and establishing long-term persistence. Thus, quasispecies diversity may serve as a critical virological determinant distinguishing acute, self-limiting infections from those that progress to chronicity.

### Immune evasion by HEV

**Figure 3:**
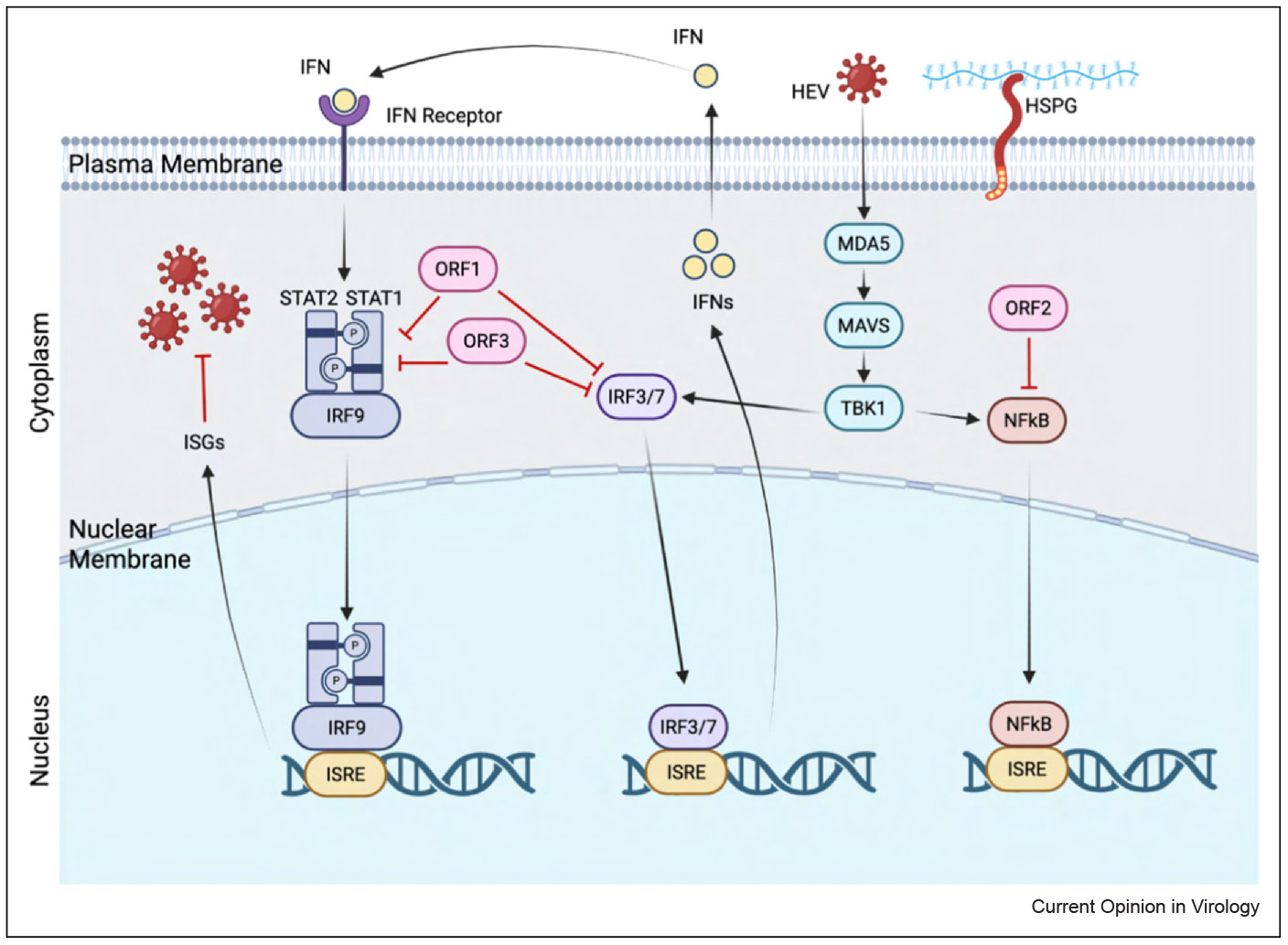
IFN signaling and regulation mechanisms of ORF1, ORF2, and ORF3 during HEV infection. During infection, HEV is recognized by host receptors such as MDA5, which leads to a series of downstream cascades, triggering the generation of IFN molecules. These IFNs can bind to plasma membrane receptors of host cells, prompting STAT1 and STAT2 phosphorylation and eventual transcription and upregulation of ISGs. Both ORF1 and ORF3 have inhibitory effects on the phosphorylation of STAT1 and IRF3/7, which limit the transcription of ISGs and the production of IFNs, respectively. ORF2 inhibits NF-kB, a downstream protein complex that is activated in the presence of HEV (the figure was generated with Biorender.com).

Recent studies have highlighted the unique egress pathway of HEV, wherein the virus is released from infected cells in a quasi-enveloped form (eHEV) through the MVB/exosome pathway [13]. The ORF3 protein plays a critical role in this process by interacting with components of the endosomal sorting complexes required for transport machinery to mediate budding into MVBs [14,15]. These quasi-enveloped virions are cloaked in host-derived membranes, helping HEV evade neutralizing antibodies in the bloodstream. However, upon secretion into bile, the quasi-envelope is stripped, and the virus is shed in the feces as nonenveloped particles that are more immunogenic [24]. While quasi-envelopment may transiently shield HEV from humoral immunity, it does not appear sufficient to support long-term immune evasion or persistence (Figure 3). In contrast to HBV and HCV, where robust and multifaceted immune evasion mechanisms contribute to chronicity, the limited immune shielding offered by eHEV likely delays but does not prevent immune recognition and clearance. Thus, HEV’s egress strategy represents a modest and temporary immune evasion tactic that may influence early viral kinetics but not long-term outcomes.

## Host immune response to HEV

The host immune response plays a pivotal role in determining the clinical trajectory of HEV infection. In immunocompetent individuals, the immune system generally mounts a rapid and effective response that facilitates viral clearance, consistent with the virus’s typically self-limiting course. However, chronic HEV infection has been increasingly recognized in immunosuppressed populations **as discussed earlier** [9-11]. This dichotomy underscores the crucial influence of both innate and adaptive immunity in shaping the outcome of HEV infection. In this section, we explore the key features of immune activation in response to HEV and contrast them with the immune landscapes of HBV and HCV infections.

### Innate immune response

In the case of HEV, the innate immune response is typically rapid and effective, playing a critical role in the self-limiting nature of infection observed in immunocompetent individuals. Studies using hepatocyte-derived cell lines and primary or primary-like human hepatocytes have demonstrated that HEV infection triggers robust production of type III interferons (IFNs), particularly IFN-λ, alongside activation of interferon-stimulated genes (ISGs) such as OAS1, MX1, and IFITM3 [25-27]. These ISGs exert antiviral effects by targeting multiple stages of the HEV life cycle, thereby restricting viral replication and facilitating viral clearance.

In contrast to HEV’s cytoplasmic replication, which makes its RNA accessible to innate immune sensors, HBV is often considered a ‘stealth virus’ due to its ability to avoid innate immune detection [28,29]. HBV evades recognition by encapsulating its DNA and replicating via reverse transcription within nucleocapsids, minimizing the accumulation of RNA intermediates in the cytoplasm. Additionally, HBV proteins can actively suppress innate signaling pathways [30]. As a result, type I and III IFN responses are minimal or undetectable in both acute and chronic HBV infections [28,29], especially in infected hepatocytes, allowing the virus to persist with limited activation of the host’s innate immune defenses.

HCV, while triggering pattern recognition receptor signaling, has evolved potent mechanisms to subvert it. The HCV NS3/4A protease cleaves mitochondrial antiviral signaling (MAVS) protein at the mitochondrial membrane, effectively disabling RIG-I-mediated signaling [31]. NS3/4A also targets TIR domain-containing adaptor molecule 1 (TICAM1 or TRIF), the adaptor molecule downstream of toll-like receptor 3 (TLR3), further impairing the production of type I IFNs [32]. These immune evasion strategies blunt the host’s antiviral response even as it is being initiated, contributing to HCV’s high rate of chronicity and eventual immune exhaustion.

HEV does exhibit some limited capacity to modulate host immunity, primarily during early infection. However, its immune evasion mechanisms are modest compared to those of HBV and HCV [33]. The multifunctional ORF3 protein plays a central role in this process (Figure 3). Beyond facilitating virion egress, ORF3 has been shown to interfere with signal transducer and activator of transcription 1 (STAT1) phosphorylation [34], dampening type I IFN signaling. ORF3’s interactions with the microtubule network [35] and its ion channel activity [36] may further affect intracellular trafficking and cellular stress pathways. The ORF1 polyprotein also contributes to immune modulation. The methyltransferase and papain-like cysteine protease (PCP) domains have been reported to inhibit RNA sensor RIG1 (RIG-I) signaling and downstream interferon regulatory factor 3 (IRF3) activation [37,38]. In addition, the macro domain within ORF1 may regulate host ADP-ribosylation pathways [39], which influence inflammation and stress responses. Nonetheless, these effects are likely transient and are weaker than HCV’s protease-mediated suppression or HBV’s stealth-like replication (Table 2).

In summary, HEV possesses relatively mild immune evasion strategies. Its ability to transiently suppress IFN responses via ORF1 and ORF3 may support early replication and viral dissemination, but it lacks the potency required for long-term persistence. Rather than fully subverting host immunity, HEV appears to adopt a strategy of moderate interference, facilitating viral clearance while minimizing immune-mediated liver injury in most immunocompetent individuals.

### Adaptive immune response

The adaptive immune response plays a central role in the resolution of viral hepatitis and in determining the progression to chronicity. In the case of HEV, adaptive immunity — comprising both T-cell and B-cell responses — is typically vigorous and effective, contributing to the clearance of infection in immunocompetent individuals [40-42]. In contrast, chronicity in HBV and HCV infections is frequently associated with impaired or exhausted adaptive immune responses [43-45] and the presence of persistent viral reservoirs.

#### T-cell responses and exhaustion profiles

**Figure 4:**
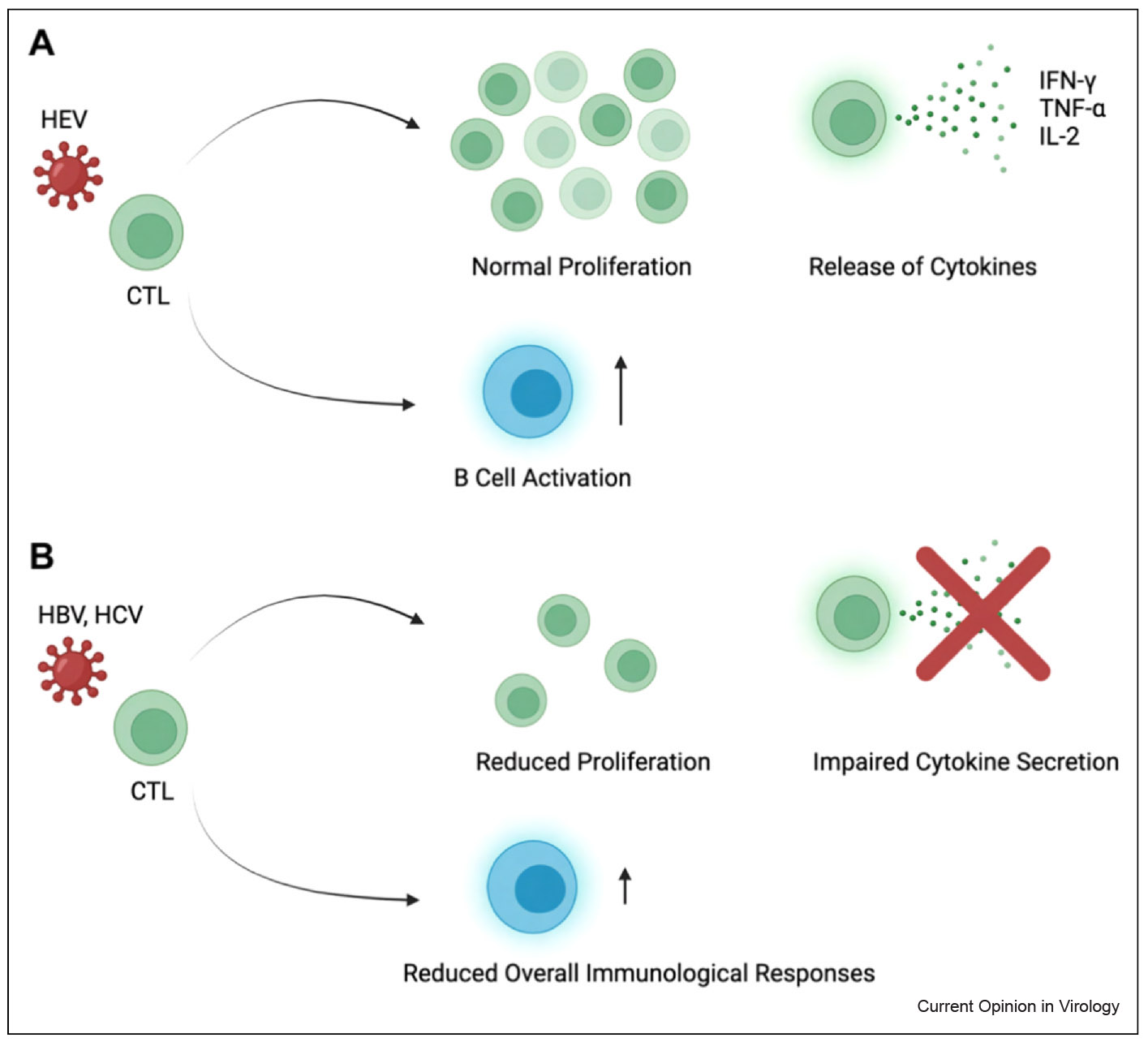
T cell responses in HBV, HCV, and HEV infection. **(a)** In HEV infection, cytotoxic T lymphocytes (CTLs) maintain normal immunological functionalities, such as proliferation of T cells, production of cytokines, and promotion of B cell activation. **(b)** In HBV and HCV infection, exhausted CTLs exhibit dysfunctional characteristics, such as reduced proliferation, impaired cytokine secretion, and decreased immunological responses (the figure was generated with Biorender.com).

During acute HEV infection, virus-specific CD8^+^ cytotoxic T lymphocytes (CTLs) are rapidly activated and play a crucial role in eliminating infected hepatocytes [40]. Simultaneously, CD4^+^ helper T cells support the priming and maintenance of CTL responses and promote B-cell activation and immunoglobulin class switching [46]. These T-cell responses in HEV-infected individuals are generally polyfunctional — characterized by robust production of cytokines such as IFN-γ, TNF-α, and IL-2 [40,47] — and typically lack the functional exhaustion phenotypes observed in chronic viral infections (Figure 4).

By contrast, HBV- and HCV-specific T-cell responses in chronically infected patients are often weak, dysfunctional, or fully exhausted [43-45]. Exhausted T cells exhibit high expression of inhibitory receptors, such as PD-1, CTLA-4, and TIM-3, and show reduced proliferative capacity, impaired cytokine secretion, and diminished cytotoxic function [48]. This exhaustion phenotype severely compromises viral control, allowing HBV and HCV to persist for decades. In HBV, chronic antigen exposure, driven in part by stable cccDNA-mediated transcription and a tolerogenic hepatic environment, further impairs T-cell function. In HCV, continuous viral replication and rapid antigenic variation contribute to immune escape and progressive T-cell exhaustion.

In HEV infection, T-cell exhaustion is rarely observed in immunocompetent individuals, likely due to the transient nature of antigen exposure and the typically short duration of infection. Even in cases of chronic HEV infection among immunosuppressed individuals, the degree of T-cell exhaustion appears more limited and less entrenched than in chronic HBV or HCV infections [40,49,50]. This fundamental immunological difference highlights a key factor favoring spontaneous viral clearance in the majority of HEV cases.

#### Humoral immunity and memory

Humoral responses also play a critical role in HEV clearance. HEV-specific IgM antibodies are detectable during acute infection and are often used diagnostically. These are followed by the emergence of long-lived IgG antibodies, which provide protection against reinfection [51]. Vaccine studies in both humans and animal models have demonstrated that the antibody response is protective, and neutralizing antibodies target epitopes on the ORF2 capsid protein [52,53].

In HBV, although neutralizing antibodies (primarily targeting HBsAg) are protective and form the basis of the effective HBV vaccine, their appearance in natural infection is often delayed or absent in chronic cases [50,54]. In HCV, the role of humoral immunity is less clearly defined, as the virus can rapidly mutate to escape neutralizing antibodies [55,56], and spontaneous clearance is more often associated with cellular immune responses [23].

#### Viral reservoirs and persistence

Another critical factor shaping the outcome of adaptive immune responses is the virus’s ability to establish stable intracellular reservoirs. In the case of HBV, the virus forms cccDNA within the nuclei of hepatocytes. This episomal DNA serves as a long-lasting template for viral mRNA and antigen production, remaining largely refractory to current antiviral therapies and posing a major barrier to viral eradication [16]. Moreover, HBV DNA can integrate into the host genome [57], leading to continued expression of viral proteins even in the absence of active viral replication, which contributes to both immune evasion and oncogenesis [58,59].

HCV, while it does not integrate into the host genome or form a nuclear reservoir like cccDNA, achieves persistence through continuous cytoplasmic replication [60,61] and its high genetic variability [62]. This genetic diversity allows the virus to evade adaptive immune recognition by rapidly generating escape mutants. Infected hepatocytes sustain long-term production of infectious virions, perpetuating chronic inflammation and liver damage unless cleared by antiviral therapy.

In sharp contrast, HEV does not establish any known stable intracellular reservoir (Table 1). Its replication is entirely confined to the cytoplasm, and the absence of a DNA intermediate precludes integration into the host genome. Once the immune system eliminates infected cells, there is no latent or residual form of the virus to trigger reinfection. This absence of a persistent reservoir aligns with HEV’s typically self-limiting course in immunocompetent individuals and underscores the capacity of the adaptive immune system to effectively resolve infection.

## Chronicity in special populations

While HEV infection is typically acute and self-limiting in immunocompetent individuals, an important exception arises in immunocompromised populations [9-11]. In these individuals — including solid organ transplant recipients, hematologic malignancy patients undergoing chemotherapy, individuals with advanced HIV/AIDS, and those on long-term immunosuppressive therapy — HEV infection can become chronic, with detectable viral RNA persisting in blood and stool for more than six months.

Chronic HEV infection was first reported in 2008 in a liver transplant recipient [63], and since then, accumulating evidence has shown that up to 60% of organ transplant recipients infected with genotype 3 HEV may fail to clear the virus without intervention [64,65]. This striking contrast highlights the essential role of a functional immune system — particularly T-cell–mediated immunity — in determining infection outcome.

The mechanisms underlying this susceptibility in immunocompromised hosts reflect failures in both innate and adaptive responses. Impaired production of IFNs, defective activation of dendritic cells, and reduced CTL activity all compromise early viral control. In transplant recipients, immunosuppressive agents such as tacrolimus directly inhibit T-cell proliferation and function, thereby compromising the host’s ability to clear HEV infection [66,67]. Notably, in over 30% of cases, reducing immunosuppressive therapy has been associated with improved viral clearance, underscoring the critical role of drug-specific modulation of immune responses in determining infection outcomes [67].

Furthermore, HIV-infected individuals with low CD4^+^ T-cell counts also show a higher risk for chronic HEV infection [68], again implicating cellular immunity in resolving the infection. In these populations, HEV-specific T-cell responses are often weak or undetectable, and humoral responses (e.g. IgG seroconversion) may be delayed or absent, compounding the risk for chronicity.

Another population of concern includes patients with pre-existing liver disease — particularly those with HBV or HCV co-infection — who may experience acute-on-chronic liver failure upon HEV superinfection [69,70]. Although these cases are often still self-limited in duration, the hepatic decompensation and mortality risk are substantially elevated, suggesting that liver reserve and immune regulation act as critical modulators of HEV disease severity.

## Conclusion and perspectives

The unique clinical trajectory of HEV infection — characterized by its typically acute, self-limiting course in immunocompetent hosts — stands in stark contrast to the often chronic and progressive nature of HBV and HCV infections. This disparity arises from a confluence of viral and host determinants that shape the outcome of infection.

From the host perspective, HEV elicits robust innate immune responses, particularly via type I and III IFNs and ISG activation. Its adaptive immune profile — marked by polyfunctional T cells and durable antibody responses — further ensures clearance in most healthy individuals. In contrast, the chronicity of HBV and HCV is closely linked to their capacity to subvert or exhaust host immune responses, leading to immune tolerance and persistent viremia. Nevertheless, the story is different in special populations. In immunosuppressed individuals, particularly those with compromised T-cell function, HEV can establish chronic infection, sometimes progressing to liver fibrosis and cirrhosis. These cases underscore the critical dependence of HEV clearance on effective host immunity and highlight the need for vigilant monitoring and therapeutic intervention in vulnerable populations.

Despite major strides in understanding HEV virology and immunobiology, several questions remain. For example, the full spectrum of HEV’s interactions with host innate immune sensors remains poorly understood. Whether certain viral genotypes exhibit differential immunomodulatory effects or tissue tropism remains an open question. Additionally, while a licensed vaccine (Hecolin) exists in China [71], its broader use and efficacy across diverse genotypes and immunocompromised groups require further investigation. Therapeutically, management of chronic HEV in immunocompromised patients typically starts with reducing immunosuppression when feasible, followed by ribavirin monotherapy as first-line therapy [72]; pegylated IFN-α can be effective in selected liver-transplant recipients but is generally avoided in other solid-organ transplants because of rejection risk [73]; sofosbuvir (alone or added to ribavirin) has shown mixed and often unsustained antiviral effects in small series and case reports, and remains investigational [74,75].

The development of more robust animal models and *in vitro* systems will be essential to dissect these mechanisms. Moreover, defining correlates of protective immunity will aid in vaccine development and inform strategies to manage chronic HEV in transplant and immunosuppressed populations.

In summary, HEV represents a model of viral clearance through balanced host–pathogen interactions. By contrasting it with HBV and HCV, we gain deeper insights into the principles that govern viral persistence and resolution — insights that may inform broader strategies for antiviral therapy and vaccine design across hepatitis viruses.

## Figures and Tables

**Table 1:** Comparison between HBV, HCV, and HEV.

Feature	Hepatitis B (HBV)	Hepatitis C (HCV)	Hepatitis E (HEV)
Genome	Partially double-stranded circular DNA	Single-stranded positive-sense RNA	Single-stranded positive-sense RNA
Replication strategy	Reverse transcription of an RNA intermediate (pre-genomic RNA)	RNA replication facilitated by viral replication complexes (nonstructural proteins)	Asymmetric replication via a negative-sense RNA intermediate
Long-lived nuclear genome form	Yes (covalently closed circular DNA - cccDNA)	No	No
Integration into host genome	Yes	No	No, though the genome can acquire insertions from host genes
Transmission	Primarily through blood, semen, and other body fluids. Common modes include birth (mother to baby), sexual contact, sharing needles, and exposure to blood or open sores.	Primarily through exposure to infectious blood. Common modes include sharing needles or other drug equipment, birth (mother to baby), healthcare-associated outbreaks (due to poor infection control), and, less commonly, through sexual contact or sharing personal items like razors or toothbrushes.	Primarily through the fecal-oral route, usually by consuming water or food contaminated with infected stool. In developed countries, consumption of undercooked pork, venison, or shellfish can also lead to infection.
Chronicity	Can lead to chronic infection (~5%), especially in infants (90%).	Can lead to chronic infection in most infected adults (70 - 85%).	Usually causes an acute, self-limiting infection, with most people recovering fully. Chronic infection is rare and typically only occurs in immunocompromised individuals.
Severity	Severity can range from mild to severe, and chronic infection can lead to serious liver problems like cirrhosis and liver cancer.	Severity can range from mild illness to serious, lifelong illness, including liver cirrhosis and cancer.	Typically resolves within a few weeks, but can be severe, especially in pregnant women. Acute liver failure is a rare but potentially fatal complication.
Genetic variability	Moderate; genotypic diversity observed.	High; extensive quasispecies complexity.	Low; genetically stable genome?
T cell dysfunction	Pronounced CD8^+^ T cell exhaustion	Pronounced, especially in the chronic phase	Minimal; robust T cell responses observed in immunocompetent hosts

**Table 2:** Comparison of interferon responses and immune evasion in HBV, HCV, and HEV.

Feature	Hepatitis B (HBV)	Hepatitis C (HCV)	Hepatitis E (HEV)
IFN induction	Minimal or undetectable	Strong early induction but suppressed by viral proteins	Robust induction, especially type III IFNs (IFN-λ)
IFN types involved	Type I and III interferons minimal	Mainly type I interferons, some type III	Mainly type III interferons (IFN-λ), some type I
Mechanism of IFN Evasion	- DNA encapsulated in nucleocapsids prevents RNA sensing - Viral proteins actively suppress IFN pathways	- NS3/4A protease cleaves MAVS and TRIF - Suppresses IFN production and signaling	- ORF3 inhibits STAT1 phosphorylation - ORF1 inhibits RIG-I signaling and IRF3 activation
Effect on IFN Response	Suppressed, allowing viral persistence	Suppressed, leading to chronic infection and immune exhaustion	Moderately suppressed, allowing effective early immune response and viral clearance
Immune Evasion Strength	Strong, ‘stealth’ virus.	Strong, protease-mediated suppression	Mild, transient immune modulation.

## Data Availability

Data will be made available on request.
